# Sodium Intake Estimation in Hospital Patients Using AI-Based Imaging: Prospective Pilot Study

**DOI:** 10.2196/48690

**Published:** 2024-02-16

**Authors:** Jiwon Ryu, Sejoong Kim, Yejee Lim, Jung Hun Ohn, Sun-wook Kim, Jae Ho Cho, Hee Sun Park, Jongchan Lee, Eun Sun Kim, Nak-Hyun Kim, Ji Eun Song, Su Hwan Kim, Eui-Chang Suh, Doniyorjon Mukhtorov, Jung Hyun Park, Sung Kweon Kim, Hye Won Kim

**Affiliations:** 1 Hospital Medicine Center Seoul National University Bundang Hospital Seongnam-si Republic of Korea; 2 Department of Internal Medicine Seoul National University Bundang Hospital Seongnam-si Republic of Korea; 3 Department of Internal Medicine Seoul National University College of Medicine Seoul Republic of Korea; 4 Center for Artificial Intelligence in Healthcare Seoul National University Bundang Hospital Seongnam-si Republic of Korea; 5 Department of Nursing Seoul National University Bundang Hospital Seongnam Republic of Korea; 6 Biomedical Research Institute Seoul National University Hospital Seoul Republic of Korea; 7 Department of Medicine Seoul National University College of Medicine Seoul Republic of Korea; 8 LOAPI-Healthcare AItheNutrigene Seongnam-si Republic of Korea

**Keywords:** artificial intelligence, AI, image-to-text, smart nutrition, eHealth, urine, validation, AI image, food AI, hospital, sodium intake, pilot study, imaging, diet, diet management, sex, age

## Abstract

**Background:**

Measurement of sodium intake in hospitalized patients is critical for their care. In this study, artificial intelligence (AI)–based imaging was performed to determine sodium intake in these patients.

**Objective:**

The applicability of a diet management system was evaluated using AI-based imaging to assess the sodium content of diets prescribed for hospitalized patients.

**Methods:**

Based on the information on the already investigated nutrients and quantity of food, consumed sodium was analyzed through photographs obtained before and after a meal. We used a hybrid model that first leveraged the capabilities of the You Only Look Once, version 4 (YOLOv4) architecture for the detection of food and dish areas in images. Following this initial detection, 2 distinct approaches were adopted for further classification: a custom ResNet-101 model and a hyperspectral imaging-based technique. These methodologies focused on accurate classification and estimation of the food quantity and sodium amount, respectively. The 24-hour urine sodium (UNa) value was measured as a reference for evaluating the sodium intake.

**Results:**

Results were analyzed using complete data from 25 participants out of the total 54 enrolled individuals. The median sodium intake calculated by the AI algorithm (AI-Na) was determined to be 2022.7 mg per day/person (adjusted by administered fluids). A significant correlation was observed between AI-Na and 24-hour UNa, while there was a notable disparity between them. A regression analysis, considering patient characteristics (eg, gender, age, renal function, the use of diuretics, and administered fluids) yielded a formula accounting for the interaction between AI-Na and 24-hour UNa. Consequently, it was concluded that AI-Na holds clinical significance in estimating salt intake for hospitalized patients using images without the need for 24-hour UNa measurements. The degree of correlation between AI-Na and 24-hour UNa was found to vary depending on the use of diuretics.

**Conclusions:**

This study highlights the potential of AI-based imaging for determining sodium intake in hospitalized patients.

## Introduction

### Overview

A low-salt diet is prescribed for patients with cardiovascular, kidney, or liver diseases. In these patients, sodium intake regulation is essential. High salt intake is a key modifiable risk factor for these diseases. Thus, monitoring the dietary salt intake of patients provides patients and clinicians with valuable information on dietary salt reduction advice [1].

For therapeutic purposes, the salt intake of hospitalized patients is assessed either indirectly by the prescription order of diet and food consumption questionnaires or directly from repeated 24-hour urinary sodium (UNa) excretion collections [2]. However, these methods have several limitations. Discrepantly with the prescribed diet order, the actual food intake of patients may vary because of poor compliance. Inaccurate answers provided during questionnaire surveys may result in biased results. Direct access to repeated 24-hour UNa collection, which is the standard method of salt intake marking, is an inconvenient and costly process because samples are to be sent to a laboratory, and flame photometry is typically required [3]. Furthermore, this method is inherently impractical because of the dependence on patient compliance and variability between collections.

For inpatients, a quick evaluation of salt intake would enable real-time advice and subsequent application to the next diet regimen. Therefore, a tool to objectively quantify a patient’s dietary intake and repetitively evaluate the sodium content of the diet of hospitalized patients is required.

Artificial intelligence (AI) technology has enabled image-based analyses of nutrition and ingredients. Picture-to-Amount, a deep learning architecture using a cross-modal image-to-text retrieval system, can predict the number of ingredients in a given food image [4]. Technology-assisted dietary assessment relies on AI to accurately group food pictures and measure food credit by assessing a cellphone food record [5]. AI models for dietary assessment have been verified for reproducibility and validity. A large-scale population survey verified the accuracy of one such AI model for nutrient analysis [6]. Carter et al [7] conducted a study to compare the nutritional intake recorded in the smartphone app called “My Meal Mate” and the nutritional intake using the 24-hour recall method. Ahmed et al [8] divided participants of their study into 2 groups, namely a meal diary group and a tablet application use, and compared their nutrient intakes [8]. A fully automatic monitoring system of nutrient intake by hospitalized patients was presented for medical use by processing Red Green Blue (RGB) depth image pairs before and after meal consumption using AI-based estimation [9]. However, most studies have focused on classifying food or identifying the content of protein, fats, and carbohydrates, whereas studies on specific nutrients such as salt are scarce. To further improve the assessment and monitoring of the salt content of inpatients’ diets, the validation of accurate estimation of the diet and consumption of salt by patients is still required.

### Objectives

We evaluated whether sodium intake could be determined through AI methods using food photographs and known nutrition information in hospitalized patients. We also determined if it is significant to compare sodium intake with 24-hour UNa, which is the gold standard for measuring sodium intake.

## Methods

### Study Design and Population

This single-center prospective study was conducted from August to November 2021 and involved 54 hospitalized patients, recruited from the hospitalist-run acute care unit as well as nephrology and urology departments. The following criteria were used for inclusion: (1) adult patients aged 19 years or older, (2) patients who agreed to take photographs before and after meals, and (3) patients who were prescribed 10 g of salt (4 g of sodium) in their diet. Patients who were unable to eat because of conditions such as respiratory arrest requiring tracheal intubation, cardiac arrest, acute coronary syndrome or life-threatening arrhythmias, failure of more than 2 organs, recent trauma, or burns of the neck and face were excluded. People with alcohol addiction and pregnant patients were also excluded. Previous medical history, demographics, and laboratory data were retrieved from electronic medical records.

#### Nutrient Information and Acquisition of Food Images

**Figure 1:**
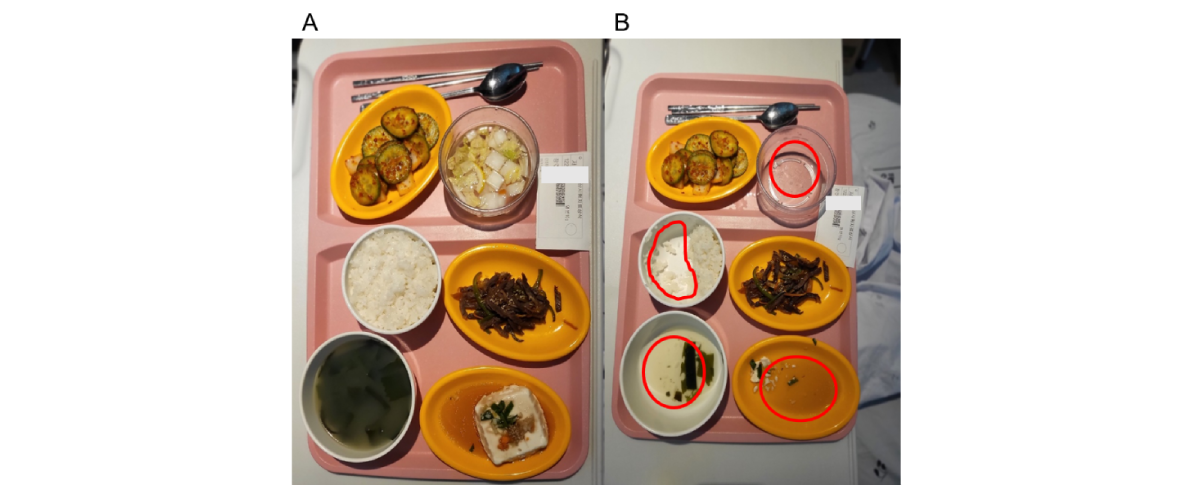
Food photographs taken (A) before and (B) after meal consumption for sodium intake measurement. The red track indicates the recognition of the amount of food intake.

A novel and dedicated image database was developed because models pretrained using the data of different regions and countries could not be used. Hence, curating a local food data set was necessary, particularly for measuring sodium intake in hospitalized patients. A 3-month diet was predetermined before the study was initiated. The Seoul National University Bundang Hospital Nutrition Department provided all the meals and nutrient information. To establish the new data set and salt estimation algorithm, photographs of cooked food, plates, and spoons from the selected menu were collected. The researchers took photographs of the patients’ meals before and after consumption for a day and prohibited the patients from eating snacks or any outside food, except for water (Figure 1A and 1B).

**Figure 2:**
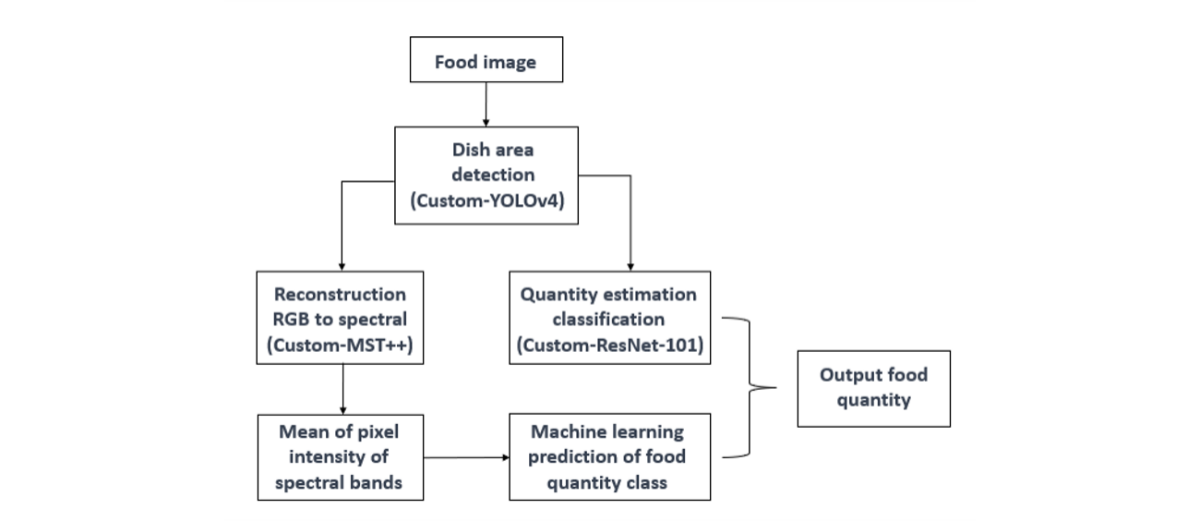
Main architecture of the hybrid model for food quantity estimation for and sodium amount estimation. RGB: Red Green Blue.

#### Food Image Analysis—Sodium Intake Measurement with AI (AI-Na)

**Figure 3:**
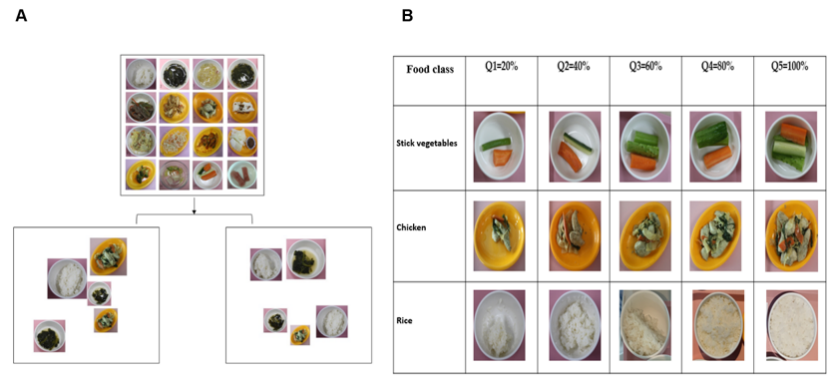
Sodium intake measurement using the artificial intelligence–based method. (A) Data generated after converting single images into multidish images; (B) Dataset for the convolutional neural network. 'Q' denotes quantity in the classification of food classes by portion size.

We used the hybrid model for sodium intake measurement based on food image analysis. This model incorporates the You Only Look Once, version 4 (YOLOv4) model’s detection of the image areas and uses 2 classification approaches—Custom-101 and Hyperspectral imaging—to predict the food quantity estimation for sodium amount (Figure 3A and 3B). YOLOv4 has been used in several previous studies to detect and classify the food and dish areas in the images [10,11]. Multidish images were generated by cropped images using the boundary-box area to create a data set containing images with different sizes. Figure 3 shows data generated after converting single images into multidish images. A data set for a convolutional neural network with one kind of food was placed in dishes of different shapes. Then, all the food images were rotated to different angles, and the light and sharpness of these images were adjusted (Figure 3B). For the estimation of food quantity from the images, we predominantly used convolutional neural networks, with ResNet-101 being our primary model of choice. In our pursuit to select the optimal classification model, several alternative architectures were also evaluated. This included experimenting with models such as MobileNetV2, ResNet-18, Wide-ResNet-50, and InceptionV3. After rigorous testing and evaluation, ResNet-101 demonstrated superior performance, affirming its selection for our research objectives. The amount of food remaining was estimated, and the calorie and nutrition contents were estimated [12,13]. The food photographs were color-based images, and a hyperspectral image was used to clarify the differences between colors and improve image quality. Hyperspectral images have rich information and show superior performance when used for feature identification based on pixel intensity [14,15]. In our study, we used Custom-MST++ to facilitate the conversion of standard RGB images into hyperspectral images. Although a conventional RGB image is composed of 3 channels (ie, red, green, and blue), the reconstructed hyperspectral image boasts an enhanced structure, encompassing 31 distinct bands. This transition from a trichannel format to a multiband representation allows for a more nuanced and detailed analysis, critical for our research objectives. The following steps must be performed when using hyperspectral images for estimating food quantity: (1) reconstruction of the hyperspectral image from the RGB image, (2) preprocessing of the hyperspectral image thus obtained, (3) calculation of the pixel intensity per spectral band, and (4) classification with of the food quantity using random-forest regression of food quantity. Therefore, by using the aforementioned method and procedures, the types and quantities of food that the patients consumed were classified and estimated from the food images. In our research, our foremost goal was to devise an algorithm capable of estimating sodium intake solely from images. Initially, our challenge was to accurately discern the volume or weight of the food items depicted in these images. Leveraging cutting-edge AI methodologies, we crafted a model adept at both recognizing and quantifying various food items. As part of this implementation process, we used information regarding the selected menu’s nutrients, type of food, and amount of food based on the protocol of the hospital nutrition department. Once we established the food quantity, we cross-referenced it with the nutritional data pertinent to the identified items. By combining this food quantity data with the nutritional profiles, our algorithm adeptly calculated the sodium proportion in the given dishes, ultimately yielding the sodium intake value, which we have denoted as AI-Na (unadjusted). Subsequent adjustments were made to the algorithm by subtracting sodium content from administered fluids, resulting in a refined AI-Na (adjusted; Figure 2).

### The 24-Hour UNa Collection as a Reference Value for Sodium Intake

The gold standard for estimating dietary sodium intake is the 24-hour UNa value [16]. Participants were asked to collect all urine during a 24-hour period, starting with the first urine sample on the morning of the day when they took the food photos and concluding with the second urine sample on the following morning. The urine aliquots were stored at –20 °C before transportation to the certified laboratory. An ion-selective electrode method (Modular DPE chemistry; Roche Diagnostics) was used to measure urinary sodium and potassium levels. The urinary creatinine (UCr) level was measured using the Jaffe reaction (kinetic colorimetric assay; Roche Diagnostics). The urine samples were excluded if any of the following were observed: (1) total volume of urine during the 24-hour period was <500 cc, (2) UCr was <0.6 g/day in men and <0.4 g/day in women, and (3) the self-reported spillage was more than 30 cc [17,18].

### Statistical Analysis

The Mann-Whitney test and Brand–Altman method were performed to determine the extent to which the AI-Na values matched the 24-hour UNa as reference values. However, because diuretics affect the 24-hour UNa, we divided the participants into diuretic and nondiuretic groups for the analysis. As kidney function can also affect the 24-hour UNa, a regression equation using sodium intake, 24-hour UNa, and estimated glomerular filtration rate (eGFR) was developed, and an interaction term was used to evaluate the role of diuretics. The interaction term was used rather than the dose, depending on the use of diuretics, as the sample was too small to evaluate the relationship between diuretic dose and 24-hour UNa. In the baseline characteristics of the study population, continuous variables were expressed as median values, and categorical variables were described as frequencies and percentages. We considered 2-sided *P* values <.05 to be statistically significant. Statistical analyses were performed using the R 4.1.0 (R foundation for Statistical Computing).

### Ethical Considerations

The study followed the general treatment policy for the underlying disease, and patients did not go beyond the scope of the standard treatment, except for capturing photographs of the prescribed meal. The study protocol complied with the Declaration of Helsinki and was approved by the Institutional Review Board of the Seoul National University Bundang Hospital (B2108-701-302). Written informed consent was obtained from all the patients.

## Results

### Estimating Sodium Intake From Food Images—Quantifying Results of Food Quantity Estimation

We collected 20,000 images for food quantity estimation and 1500 hospital images with sodium amount metadata. Table 1 and Table 2 show the results of food area detection and classification. In the context of our research, when we refer to YOLO versions, such as YOLOv3, YOLOv3-tiny, YOLOv3-tiny3l, YOLOv4, and YOLOv4-tiny, an important part of the highlight is that our choice of YOLOv4 signifies our preference for the best training and testing mean average precision among these versions. The decision was made after rigorous evaluation and comparative analysis, ensuring optimal performance for our specific use case. Moreover, similar to choosing the ResNet-101 architecture for its distinct advantages in certain applications, we used 5 alternative CNN models to get the best accuracy of classification. ResNet-101 got the highest *F*_1_-score and a lower train and validation loss in 50 epochs. Training and validation losses quantify the model’s prediction error. Lower values indicate higher accuracy and better generalization to new data. These losses are measured as dimensionless quantities derived from the chosen loss function, such as cross-entropy for classification tasks. Epoch count, such as the 50 used in our experiments, represents the total number of times the learning algorithm processes the entire data set, a critical factor in optimizing the model’s learning curve and preventing overfitting or underfitting.

**Figure 4:**
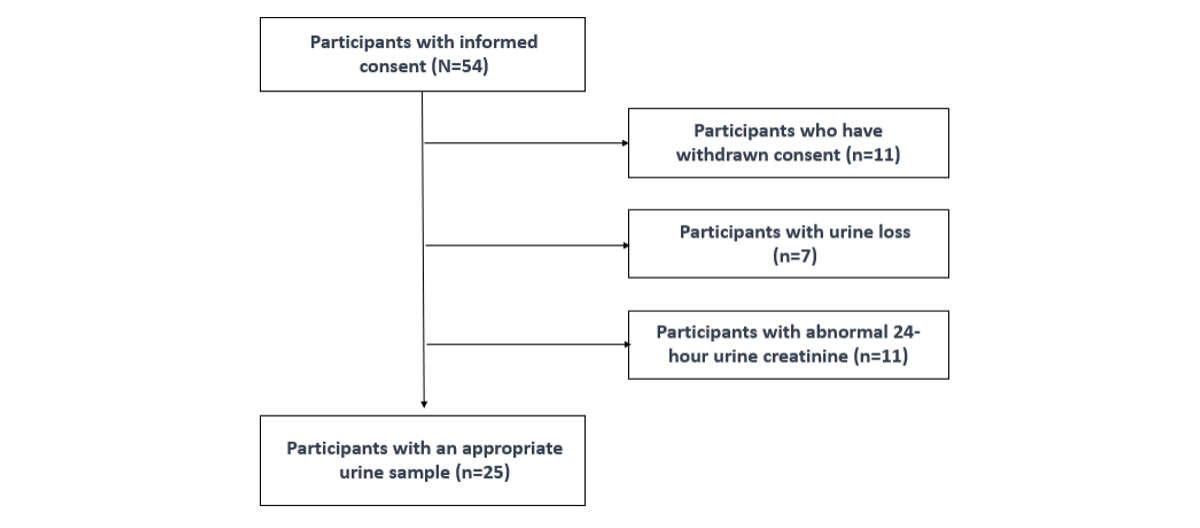
Flow diagram of the study population.

Of the 54 participants enrolled in this study, 11 withdrew their consent because they could not wait for the photographs to be taken before the meal and because their general health deteriorated. Seven participants were excluded due to incomplete urine collection, and 11 were excluded due to inaccurate urine collection based on the 24-hour UCr value. Finally, the data of the 25 selected participants were analyzed (Figure 4).

The median age was 64 (IQR 53-74) years, and 68% (n=17) were men. The median values of serum creatinine and serum sodium were 1.0 (IQR 0.8-1.6) mg/dl and 138 (IQR 135.0-140.0) mEq/ml, respectively. The baseline characteristics of the study participants are presented in Table 3. Because the use of diuretics affects 24-hour Una—the standard of sodium intake evaluation—the baseline characteristics were classified accordingly (Table 3). A total of 10 participants were treated with saline and parenteral nutrients. Because sodium in these fluids affects the 24-hour UNa, total intake was calculated by adding the amount of sodium administered (AI-Na [adjusted]).

The median sodium intake (AI-Na [unadjusted]) was estimated to be 1756.5 (IQR 1266.6-2273.2) mg when using the AI algorithm; further, the sodium in the fluids was included, resulting in a total sodium intake AI-Na [adjusted]) of 2022.7 (IQR 1396.2-2564.4) mg (Table 3). The 24-hour UNa was determined to be 2783.0 (IQR 1955.0-4922.0) mg. Depending on the effect of the diuretics, the value of 24-hour UNa varied and was 2599.0 (IQR 1771.0-4922.0) mg and 2921.0 (IQR 2231.0-4059.5) mg for the nondiuretic and diuretic groups, respectively (Table 4).

We compared the AI-estimated sodium intake values with those of 24-hour UNa and analyzed the degree of concordance and difference between the two.

**Table 1:** Results of food area detection.

Model name	Training mAP^a^ (%)	Testing mAP (%)	Weight size (MB)	Training time (h)
YOLOv3^b^	81.1	74	236	178
YOLOv3-tiny	90	85	33.7	65
YOLOv3-tiny_3l	87.6	77	33.7	65
YOLOv4^c^	98.9	95	245	207
YOLOv4-tiny	70	74	23	64.9
YOLOv4-tiny_3l	85.1	75	23	64.3

^a^mAP: mean average precision. Input size was 608×608, and iteration numbers were 50,000.

^b^YOLOv3: You Only Look Once, version 3.

^c^YOLOv4 was selected from multiple models listed in the Table.

**Table 2:** Classification of food quantity estimation.

Model name	Training *F*_1_-score (%)	Training loss	Validation *F*_1_-score (%)	Validation loss	Epoch	Time (h)
MobileNetv2	95	0.15	93	0.7	50	0.8
ResNet-18	96	0.12	92	0.1	50	1.2
Wide-ResNet-50	96	0.2	94	0.1	50	1.3
ResNet-101^a^	98	0.11	95.5	0.03	50	1.2
Inceptionv3	96	0.17	93	0.12	50	1.4

^a^ResNet-101 was selected from multiple models listed in the Table.

**Table 3:** Comparison of the characteristics between the diuretic and nondiuretic groups.

Characteristics	Total (N=25)	Non-diuretics (n=14)	Diuretics (n=11)
Age, median (IQR)	64 (53-74)	64.5 (43.0-72.0)	62.0 (56.5-79.5)
Sex (male), n (%)	17 (68)	12 (86)	5 (46)
BMI (kg/m^2^), median (IQR)	25.8 (23.7-28.8)	24.3 (23.7-26.3)	27.4 (26.1-29.1)
Hypertension, n (%)	16 (64)	8 (57.1)	8 (73)
Diabetes, n (%)	12 (48)	6 (43)	6 (55)
Liver cirrhosis, n (%)	7 (28)	4 (29)	3 (27)
Congestive heart failure, n (%)	4 (16)	0 (0)	4 (36)
Coronary artery disease, n (%)	3 (12)	2 (14)	1 (9)
Cerebrovascular disease, n (%)	3 (12)	1 (7)	2 (18)
Cancer, n (%)	5 (20)	4 (29)	1 (9)
Chronic kidney disease, n (%)	6 (24)	4 (29)	1 (9)
Hemoglobin (mg/dl), median (IQR)	11.1(10.0-13.3)	11.6 (10.0-13.3)	10.5 (9.7-13.2)
Sodium (mEq/L), median (IQR)	138.0 (135.0-140.0)	138.0 (133.0-140.0)	138.0 (135.0-140.0)
Potassium (mEq/L), median (IQR)	4.0 (3.7-4.4)	4.0 (3.9-4.3)	4.0 (3.6-4.4)
Serum creatinine (mg/dl), median (IQR)	1.0 (0.8-1.6)	1.0 (0.7-1.4)	1.3 (0.9-1.8)
Albumin (mg/dl), median (IQR)	3.6 (3.3-4.1)	3.6 (3.3-4.0)	3.4 (3.1-4.2)
eGFR^a^ (ml/1.73m^2^), median (IQR)	70.41 (42.57-89.55)	83.82 (52.04-98.38)	54.06 (27.77-74.43)

^a^eGFR: estimated glomerular filtration rate.

**Table 4:** Sodium input calculated with AI (AI-Na) and 24-hour urine sodium (UNa) excretion.

Sodium and urine output metrics	Participants, median (IQR)				*P* value
	Total (N=25)	Nondiuretic (n=14)	Diuretic (n=11)		
AI-Na (mg; unadjusted^a^)	1756.5 (1266.6-2273.2)	1646.4 (1162.4-2244.9)	1756.5 (1620.9-2418.8)	.37	
Sodium in the fluids (mg)	0 (0.0-177.1)	177.1 (0.0-190.8)	0 (0.0-0.0)	.98	
AI-Na (mg; adjusted^b^)	2030.0 (1396.2-2564.4)	2034.2 (1266.6-2748.9)	2022.7 (1620.9-2418.8)	.98	
Total urine output (ml/day)	1170.0 (1120.0-2210.0)	1750.0 (916.0-2468.0)	1650.0 (1210.0-1984.0)	.99	
24-hour UNa (mg/day)	2783.0 (1955.0-4922.0)	2599.0 (1771.0-4922.0)	2921.0 (2231.0-4059.5)	.81	
24-hour UCr^c^ (g/day)	0.9 (0.7-1.1)	1 (0.9-1.2)	0.8 (0.6- 0.9)	.06	

^a^Unadjusted with the amount of sodium in the administered fluids.

^b^Adjusted with the amount of sodium in the administered fluids.

^c^UCr: urine creatinine.

### The Difference and Accordance Analysis Between Sodium Intake by Image-Sodium and 24-Hour UNa

The sodium intake was measured using the 2 aforementioned methods and was 2022.7 mg in AI-Na (adjusted) and 2783.0 mg in 24-hour UNa, and the statistical significance was 0.02, indicating a statistically significant difference. Considering the effect of diuretics on UNa, the participants were divided into 2 groups: diuretic and nondiuretic, and no significant difference was noted between the 2 groups (Table 5).

The disparity between the 2 methods was not insignificant, and we assessed its impact on the concordance. Our analysis using the Bland-Altman method revealed a bias of –1106.4 mg, with a CI for the concordance limit ranging from –5468.2 mg to 3255.5 mg. Given that the corresponding bias when converted to the amount of salt is approximately 2.76 g and the concordance limit’s CI varies from 8.1 g to 13.7 g, it is challenging to draw conclusive inferences from these findings.

**Table 5:** Differences between the total sodium input values using an artificial intelligence (AI)–based method (adjusted by fluids) and 24-hour urine sodium (UNa) excretion.

Sodium intake			Difference test^a^, median (IQR)		*P* value
**Total participants**					.02
	AI-Na^b^ (adjusted^c^; mg)	2022.7 (1369.2-2564.4)			
	24-hour UNa (mg)	2783.0 (1955.0-4922.0)			
**Participants with nondiuretics**					.14
	AI-Na (adjusted^c^; mg)	2034.2 (1282.1-2689.0)			
	24-hour UNa (mg)	2599.0 (1817.0-4566.0)			
**Participants with diuretics**					.10
	AI-Na (adjusted^c^; mg)	2023.0 (1621.0-2419.0)			
	24-hour UNa (mg)	2921.0 (2231.0-4060.0)			

^a^Mann-Whiney test.

^b^AI-Na: AI-estimated sodium intake.

^c^Adjusted with the amount of sodium in the administered fluids.

### Interaction Model Using Regression Analysis

The difference between the 2 test values could be attributed to factors that affect 24-hour UNa excretion in a real-world setting, such as the use of diuretics as well as the patient’s gender, age, and renal function; this prompted us to derive a formula considering the aforementioned factors. The eGFR value using the Chronic Kidney Disease Epidemiology Collaboration equation was used as a variable, and the following regression equation was obtained using the interaction term for diuretics because gender, age, and renal function can all be calculated using eGFR (Table 6).

*24h-UNa* = 0.535 * AI-Na [adjusted]-2292.009 * I (diuretics) + 1.280 * AI-Na [adjusted] * I (diuretics) + 22.102 * eGFR

*24h-UNa* = 2.355 * AI-Na [adjusted] + 22.102 * eGFR-2292.009 (diuretic group)

*24h-UNa* = 0.535 * AI-Na [adjusted] + 22.102 * eGFR (nondiuretic group)

In this equation, “I” is the interaction term.

In this regression equation, the AI-Na (adjusted) and 24-hour UNa exhibited a strong relationship. Additionally, eGFR was significantly related to 24-hour UNa. Because the impact of the eGFR value on the 24-hour UNa is the same regardless of the use of diuretics, which was negligible in this equation, it can be assumed that 2.355 times the total sodium input (AI-Na [adjusted]) corresponds to 24-hour UNa in the diuretic group, and 0.535 of the AI-Na corresponds to the measured 24-hour UNa in the nondiuretic group.

**Table 6:** Linear regression with the interaction term between sodium intake calculated by the artificial intelligence algorithm (AI-Na; adjusted) and 24-hour urine sodium (UNa) excretion (adjusted R^2^=0.739; F=18.7; *P*<.001).

Linear regression with interaction term	Regression coefficients	SE	*P* value^a^
AI-Na (mg; adjusted^b^)	0.535	0.232	.03
Furosemide dose (mg)	–2292.009	2259.709	.32
eGFR^c^ (ml/min/1.73m^2^)	22.102	8.361	.02
AI-Na (furosemide dose; adjusted)	1.820	1.076	.11

^a^*P* value for the interaction term in the relationship.

^b^Adjusted with the amount of sodium in the administered fluids.

^c^eGFR; estimated glomerular filtration rate.

## Discussion

### Principal Findings

Our study may be the first study that compared an AI-based method with 24-hour UNa for measuring sodium intake in a real clinical field. Although the AI-Na and 24-hour UNa values were not the same, the various factors that affect the 24-hour UNa value, such as age, sex, renal function, diuretics, and even the underlying disease, cannot be ignored when using real-world data. Therefore, the 2 methods were worth evaluating using regression. The AI-Na values can be clinically considered as a significant indicator of sodium intake, although there were differences based on whether diuretics were used. Therefore, food images can be used to measure sodium intake to some extent, but this method is still inaccurate.

We calculated the sodium amounts in each test image as ground truth and used them for the AI sodium amount prediction model. Food amount served as one of the input values for Sodium amount prediction, as we used a multi-input fixture to predict sodium amount prediction and 24-hour UNa amount prediction using the collected data set from the hospital. As we collected metadata, it included food quantity, sodium amount, food classes, food intake, and patient information. The AI-based method predicted salt or sodium amount in food based on our multitask method.

Concerns regarding sodium intake have led to the development of several sodium measurement methods [16,19,20]. Numerous methods for measuring sodium intakes are available, ranging from 24-hour UNa, the most objective method, to single or multiday food records and 24-hour dietary recalls in which patients have to subjectively capture detailed information regarding the food they consumed in the past 24 hours. The 24-hour UNa method shows up to 30% within-person variability. Therefore, this method has been used to validate the accuracy of dietary measurement studies [21]. Other subjective methods for assessing sodium intake are the food diary, the recall method, and concurrent dietary questionnaires. These methods describe the portion size or weight of the food consumed; therefore, these methods may not be accurate and may yield highly variable results because they rely solely on the information that the participants provide. Therefore, 24-hour UNa is generally used for the external validation of these methods. Although dietary records and 24-hour UNa are recognized as suitable methods for measuring sodium intake, challenges such as difficulty during measurement and variability in results persist. Therefore, there is a growing demand for a more accurate and convenient method to measure sodium intake.

The rising interest in health care has led to an increased demand for nutrient and diet management. With the widespread use of smartphones, apps, and advancements in AI, there have been efforts to integrate these technologies into a dietary management system. A study was conducted to evaluate the consistency of an app designed as a calorie measurement tool for weight loss [7]. The comparison focused on the results from inputting consumed food into the app with those from a phone recording. Another study compared the food recorded in the app with a written record to demonstrate the app’s validity [8]. These studies proposed a novel approach that deviates from traditional methods but still relies on patient self-recorded information. The need for objective methods of food and nutrient intake measurement has led to the emergence of AI-based studies that have classified and quantified food intake using food photos. AI-based techniques have advanced to a level at which they can be used to classify various objects or humans in photos, including the ability to identify the food on the plate and the different types of food in the image; they can also determine the food quantity [5,11,12,22]. Furthermore, the food’s ingredients can be distinguished, and even chemical and molecular information can be obtained using hyperspectral images [4,15]. These studies have focused on healthy populations, except for one that was conducted on hospitalized patients, similar to our study [9]. This study evaluated the food quantity, calories, carbohydrates, fat, and salt intake of hospitalized patients by analyzing the photos taken before and after meals. However, the reference value was calculated using the weight and nutrition information that the hospital provided, lacking the actual measurement of nutrient intake or clinical reference. Additionally, because the aforementioned study focused on the method of analyzing food photos, the experimental setting of this study did not reflect that of a real-world clinical one. Our study, on the other hand, considered several variables of an actual clinical environment and showed that salt intake measured by a photo-based AI algorithm had a significant relationship with the gold standard of sodium intake (ie, 24-hour UNa), thereby demonstrating that AI-Na value establishes the foundation for clinical use.

Accurately measuring food intake in hospitalized patients is crucial for determining their nutritional status, disease progression, or treatment option selection. However, traditional methods for evaluating food intake, such as visual estimation of the entire or 75% of the amount and patients’ subjective evaluations, often result in inaccuracies and an overestimation of up to 15% [23,24]. Therefore, a convenient and semiautomated digital method for food intake evaluation is required, and AI-based food photographs are expected to fulfill this need.

### Strengths and Limitations

Our study’s strength lies in its evaluation of sodium intake in actual inpatients using cutting-edge technologies, food photos, and AI-based techniques as well as a comparison with established standards. Various variables of a real clinical setting were considered in this study, demonstrating the potential clinical usefulness of using AI in this domain. This study had some limitations. First, the sample size considered in this study was small. If more patients were enrolled, it would be possible to derive a more accurate formula using different variables. The second limitation was the inaccurate estimation of the food amount during food intake measurement. In addition to considering 24-hour UNa, it would have been useful if the food’s weight was considered both before and after consumption along with the corresponding nutrients. Third, the method proposed in this study failed to account for the potential loss of sodium during cooking and trimming processes, which may have further impacted the accuracy of the results.

Although we presented hyperspectral image reconstruction and employed a machine learning model for estimating food quantity, it is important to note that our approach did not directly detect sodium amounts from food images. Instead, it demonstrated a robust correlation between hyperspectral images and sodium amount estimation. This correlation provides a promising foundation for future research focused on refining methods for predicting sodium content.

In summary, our study aimed to incorporate AI into the clinical field; however, owing to limitations associated with AI, incomplete nutritional information, and the diversity of real-world treatments, comprehensive research planning is necessary for clinical use.

### Conclusions

The method of measuring sodium intake using food photos was found to be inconsistent as compared with 24-hour UNa, which is widely used in clinical settings. However, the study results have clinical significance because variables of a real-world clinical setting, such as gender, age, diuretics, and fluid treatment, were considered. The findings also suggest that the formula derived in this study may not provide accurate estimates of the absolute sodium intake. However, if additional data are collected, it may be possible to develop a more useful formula in the future.

## Abbreviations

AI: artificial intelligence
AI-Na: sodium intake calculated by the AI algorithm
eGFR: estimated glomerular filtration rate
RGB: Red Green Blue
UCr: urine creatinine
UNa: urine sodium
YOLO: You Only Look Once

## Data Availability

The data set used in this research consists of food images and nutrition information. The data set is publicly available on AI Hub, a repository for AI-related data sets, maintained by the National AI Research and Development Agency of Korea. The data set can be accessed on AI Hub [25].

## References

[1] Heeney ND, Lee RH, Hockin BCD, Clarke DC, Sanatani S, Armstrong K, Sedlak T, Claydon VE (2021). At-home determination of 24-h urine sodium excretion: validation of chloride test strips and multiple spot samples. Auton Neurosci.

[2] Reducing salt intake in populations report of a WHO forum and technical meeting. World Health Organization.

[3] Van Dam RM, Hunter D (2012). Biochemical indicators of dietary intake. Nutritional Epidemiology 3rd Edition.

[4] Li J, Han F, Guerrero R, Pavlovic V (2021). Picture-to-amount (PITA): predicting relative ingredient amounts from food Images.

[5] Kalivaraprasad B, Prasad M, Vamsi R, Tejasri U, Santhoshi M, PramodKumar A (2021). Analysis of food recognition calorie estimation using AI.

[6] Lee H, Huang T, Yen L, Wu P, Chen K, Kung H, Liu C, Hsu C (2022). Precision nutrient management using artificial intelligence based on digital data collection framework. Appl Sci.

[7] Carter MC, Burley VJ, Nykjaer C, Cade JE (2013). 'My Meal Mate' (MMM): validation of the diet measures captured on a smartphone application to facilitate weight loss. Br J Nutr.

[8] Ahmed M, Mandic I, Lou W, Goodman L, Jacobs I, L'Abbé MR (2017). Validation of a Tablet Application for Assessing Dietary Intakes Compared with the Measured Food Intake/Food Waste Method in Military Personnel Consuming Field Rations. Nutrients.

[9] Lu Y, Stathopoulou T, Vasiloglou MF, Christodoulidis S, Stanga Z, Mougiakakou S (2021). An artificial intelligence-based system to assess nutrient intake for hospitalised patients. IEEE Trans Multimedia.

[10] Redmon J, Farhadi A YOLO9000: Better, faster, stronger. CVPR.

[11] Bochkovskiy A, Wang CY, Liao HYM YOLOv4: optimal speed and accuracy of object detection. arXiv.

[12] Ciocca G, Napoletano P, Schettini R (2017). Food recognition: a new dataset, experiments, and rResults. IEEE J Biomed Health Inform.

[13] Redmon J, Farhadi A YOLOv3: an incremental improvement. arXiv.

[14] Hussain A, Pu H, Sun D (2018). Innovative nondestructive imaging techniques for ripening and maturity of fruits – A review of recent applications. Trends Food Sci Technol.

[15] Wu D, Sun D (2013). Advanced applications of hyperspectral imaging technology for food quality and safety analysis and assessment: A review — Part I: Fundamentals. Innov Food Sci Emerg Technol.

[16] McLean RM, Farmer VL, Nettleton A, Cameron CM, Cook NR, Campbell NRC (2017). Assessment of dietary sodium intake using a food frequency questionnaire and 24-hour urinary sodium excretion: a systematic literature review. J Clin Hypertens (Greenwich).

[17] John KA, Cogswell ME, Campbell NR, Nowson CA, Legetic B, Hennis AJM, Patel SM (2016). Accuracy and usefulness of select methods for assessing complete collection of 24-hour urine: a systematic review. J Clin Hypertens (Greenwich).

[18] Neupane D, Rijal A, Henry ME, Kallestrup P, Koirala B, Mclachlan CS, Ghimire K, Zhao D, Sharma S, Pokharel Y, Joseph K, Olsen MH, Schutte AE, Appel LJ (2020). Mean dietary salt intake in Nepal: a population survey with 24-hour urine collections. J Clin Hypertens (Greenwich).

[19] Bentley B (2006). A review of methods to measure dietary sodium intake. J Cardiovasc Nurs.

[20] Judge C, Narula S, Mente A, Smyth A, Yusuf S, O'Donnell MJ (2021). Measuring sodium intake: research and clinical applications. J Hypertens.

[21] Kaaks R, Riboli E, Sinha R (1997). Biochemical markers of dietary intake. IARC Sci Publ.

[22] Pouladzadeh P, Shirmohammadi S, Al-Maghrabi R (2014). Measuring calorie and nutrition from food image. IEEE Trans Instrum Meas.

[23] Kandiah J, Stinnett L, Lutton D (2006). Visual plate waste in hospitalized patients: length of stay and diet order. J Am Diet Assoc.

[24] Sullivan SC, Bopp MM, Weaver DL, Sullivan DH (2016). Innovations in calculating precise nutrient intake of hospitalized patients. Nutrients.

[25] AI Hub.

